# Supplementary material for: Genomic population structure associated with repeated escape of Salmonella enterica ATCC14028s from the laboratory into nature
Source: PLoS Genet. 2021 Sep 27;17(9):e1009820. doi: 10.1371/journal.pgen.1009820 (PMC8496778; doi:10.1371/journal.pgen.1009820)
Supplement: S1 Text — History of ATCC 14028s. History of CIP 104115. History of NCTC 12023. History of NCTC12023 David Holden. History of NCTC12023 NalR Hensel. History of NCTC12023 Gerlach. Random large HC20 clusters. Outbreaks associated with HC20 clusters. HC20 clusters of laboratory strains. NCTC 7832. HC20_5519. (DOCX) [file pgen.1009820.s001.docx]

S1 Text

Genomic island instability in HC20_373. *S. enterica* core genomes include long stretches of DNA that are particularly easy to delete because of surrounding repetitive DNA. For example, the *Salmonella* Pathogenicity Island SPI-1 is found in almost all *Salmonella*, and is also considered to be essential for invasive infections because it encodes a Type Three Secretion System (TSS3) which facilitates intracellular entry into non-phagocytic cells. SPI-1 is also thought to have been acquired by *Salmonella* before the ancestral split into *S. enterica* and *S. bongori* [1]. However, SPI-1 has been lost on occasion during cultivation or storage in the laboratory, and is also absent in some natural isolates of pathogenic serovars Senftenberg and Litchfield [2]. Salmonella Pathogenicity Island SPI-2 encodes a separate TSS3 and other genes, and is important for replication inside host cells [3]. SPI-2 is present with minor variations throughout *S. enterica* [4], and is thought to have been acquired by its ancestors after they split from *Salmonella bongori* [5]. The results in Table 1 showed that 11 ATCC 14028s derivatives and strains from laboratory infections (6.5%) had lost SPI-1, SPI-2, SPI-11 and/or the virulence plasmid whereas only 3 (0.9%) of the natural isolates had lost Pathogenicity Islands or the Virulence plasmid.

History of ATCC 14028s. Internal records at the UK’s National Collection of Type Cultures (NCTC) indicate that ATCC strain numbers 14024 and 14025 were assigned at the American Type Culture Collection (Rockville, MD, USA) in December 1960 to strains that were exchanged between the two institutions. Assuming that numerical designations were assigned in sequential order, ATCC 14028 would have been deposited at ATCC late in 1960 or early in 1961. ATCC 14028 was stored in stab culture(s) at room temperature until 2003, and subsequently preserved at -70° [6]. During this period, a mutation leading to a shortened LPS resulted in a rough subculture being designated as ATCC 14028r (rough) and a different subculture with an intact LPS being designated ATCC 14028s (smooth).

History of CIP 104115. The Collection de l’Institut Pasteur (Paris) received a sub-culture of ATCC 14028 (CDC 6516-60) in 1994 (<https://catalogue-crbip.pasteur.fr/resultatRecherche.xhtml>), and has maintained it as both freeze-dried lyophils and frozen stocks. CIP 104115 has been sold 46 times in the last six years, and in one case, the customer complained that half of the colonies were rough. The genome sequenced for this project was from a batch that was lyophilized in 2013.

History of NCTC 12023. NCTC 12023 has been maintained as a freeze-dried desiccate/lyophil (Batch 1) since it was received from ATCC as ATCC14028 in January 1987 in the context of an exchange of strains. No information is available at NCTC about its prior form of maintainance, except that it had been deposited at the ATCC by P.R. Edwards (CDC, Atlanta, GA, USA; deceased 1966), and was previously designated as CDC 6516-60.

The current online NCTC catalogue (<https://www.phe-culturecollections.org.uk/products/bacteria/detail.jsp?refId=NCTC+12023&collection=nctc>) (and archived print catalogues) list this strain as having been isolated from "Bovine, septicaemic liver", whereas the ATCC lists ATCC14028s as being isolated from "Tissue, animal - pools of heart and liver from 4-week-old chickens". There is no other known archival evidence for either claim.

The genome sequence in EnteroBase is from batch 10 of NCTC 12023 which was dried on 16th October 2015 for genome sequencing within the framework of the NCTC 3000 project ([Link to NCTC3000 website](https://www.phe-culturecollections.org.uk/collections/nctc-3000-project.aspx)). Batch 10 was sub-cultured directly from Batch 1. PacBio long-read sequencing of this batch was performed by the Wellcome Trust Sanger Institute (ENA accession code ERR1140974) and the complete genome was reconstructed from those long reads (GenBank accession code ERS836404). 100 bp Illumina short read sequencing was also performed from the same batch (ERR5525600). The long read assembly was checked and corrected with EToKi module assemble according to the Illumina short read sequencing resulting in a final complete genome which is available from EnteroBase (strain barcode SAL_FB4645AA; substrain of strain 14028s with barcode SAL_EA9729AA).

History of NCTC12023 David Holden. The date of receipt is estimated as 1995, but detailed records on its acquisition are not available. DNA was freshly extracted in 2021 from the stock culture for genomic sequencing.

History of NCTC12023 NalR Hensel. NCTC12023 accompanied Michael Hensel in 1996 when he returned to Erlangen, Germany after a post-doctoral stay with David Holden. In Erlangen, he selected a spontaneous mutant resistant to nalidixic acid (Nal^R^) in order to facilitate the selection for virulent variants after systemic mouse infections. The genome sequenced for this project was from freshly extracted DNA of that Nal^R^ mutant, and contains a T->C mutation in the QRDR region of *gyrA* (S2 Table).

History of NCTC12023 Gerlach. The original Hensel variant of NCTC12023 (pre-selection for resistance to nalidixic acid) was sub-cultured and frozen in 2003, and accompanied Roman Gerlach to Wernigerode in 2008. It was maintained as a frozen stock until its genome was sequenced and published [7]. No SNPs distinguish the non-repetitive genome of this strain from NCTC 12023 Holden.

Random large HC20 clusters. In mid-2020, we screened EnteroBase to identify HC20 clusters in order to identify natural clusters encompassing >200 genomes that had been isolated over at least 10 years. Ten such clusters were identified, and their phylogenetic structure investigated by Ninja [8] NJ trees of allelic differences in cgMLST (S4-S8 Figs). Non-repetitive SNPs were determined using EToKi [9], and used to calculate basic statistics on the pairwise-differences in SNPs between individual genomes within a cluster (Table 2). Eight of the HC20 clusters have mean pairwise SNP distances of 9.5-21.5, in part because multiple multi-genome nodes are present within the NJ tree (Table 2, S4-S7 Figs). These eight HC20 clusters are designated as Random large HC20 clusters in Table 2. Each contains a single serovar, is largely restricted to a single geographical region (U.S.A. for six and the United Kingdom for two), and the temporal spread of isolations spans one to three decades. HC20_10, 710 and 1487 are predominantly associated with a single host (Swine, Swine, and Humans, respectively) and the others span multiple hosts (Table 2). We consider these properties to define the norm for large, long-lasting HC20 clusters.

In June 2021 after completing Fig 5, we investigated the basic properties of seven, additional large HC20 clusters, resulting in the observations summarized in S4 Table. Each of the seven HC20 clusters contained multiple HC10 clusters and had been isolated over more than 15 years. Three clusters (HC20_7, HC20_13 and HC20_15) were predominantly isolated in the U.S.A. from swine, poultry and poultry, respectively whereas the other four were intercontinental and isolated from multiple hosts.

Outbreaks associated with HC20 clusters. Two of the random large clusters (HC20_4179, HC20_39803) demonstrated very low genetic diversity, with mean pairwise SNP distances of only 3.0-3.2, comparable to those of HC20_373 (Table 2). HC20_4179 included multiple HC10 clusters whereas only one HC10 cluster was found in HC20_39803. Unlike the extended chains of infections found for the eight large HC20 clusters described above, most of the genomes from HC20_4179 (S8A Fig) and HC20_39803 (S8B Fig) may have arisen from discrete food-borne outbreaks.

HC20_4179 encompasses 581 genomes of serovar Newport from North America, 97% of which were from humans (2010-2021). Almost all those genomes (94%) were concentrated in HC5_236520, and had been isolated in mid-2020 in Canada, which reported an outbreak of those dimensions of serovar Newport associated with onions imported from California [10] (S8A Fig). A concurrent outbreak from the same onion supplier also infected more than 1000 individuals in the U.S. [11], but short read sequences from the CDC are not included in EnteroBase because they lack metadata, and only few isolates from the USA in 2020 are included in S8A Fig. Interestingly, HC5_236520 also includes five genomes isolated in Canada in 2015, demonstrating that this outbreak extended over several years. In agreement, genomes from other HC5 clusters in HC20_4179 also predate 2020, indicating that this entire HC20 cluster evolved over multiple years.

HC20_39803 encompasses 222 genomes of serovar Hadar isolated from humans between 2000-2018. One genome had been isolated in 2000 in Denmark, but all others with metadata were isolated in 2015-2018 from Wyoming, South Dakota, Georgia and Ohio, or from unspecified states within the U.S. Most isolates with metadata were isolated in 2017. Literature research did not succeed in identifying a Hadar outbreak which unambiguously corresponds to HC20_39803 from this period. However, we note that Hadar was one of the predominant serovars associated with salmonellosis after exposure to household chicken flocks in 2017 in the U.S. [12].

We note that a third HC20 cluster, HC20_44730 was associated with baby milk powder from a single producer [13] in 2011-2012 (Spain and France) and 2018-2019 (France, Belgium, Luxembourg). HC20_44730 was not investigated in greater detail because EnteroBase only contained 61 genomes. However, cursory examination revealed similar clustering by local outbreak within an HC20 cluster. French and Spanish isolates from 2011-2012 differed by 10 alleles from French, Belgian and Luxembourg isolates from 2018-2019, and a third branch from 2008-2019 from France, the United Kingdom, the U.S. and the Netherlands, was present at an intermediate position within the HC20 cluster.

HC20 clusters of laboratory strains. The detailed population structure of HC373 is described within the main text. It consists of multiple genomes from laboratory stocks, laboratory infections and natural sources that are associated with a very small number of nodes (genotypes), many of which are indistinguishable from multiple other genomes from the other categories. The genotypes are tightly clustered, with no large internal branches within the cluster. We also examined the population structure of two other strains of *S. enterica* that are commonly used in the laboratory, NCTC 7832 (serovar Nottingham) and SL1344 (serovar Typhimurium).

NCTC 7832 [14], serovar Nottingham, was deposited by Joan Taylor at NCTC in 1949 [15], and since been maintained there and commercially available for purchase. NCTC 7832 is recommended for use as a quality control for growth of *Salmonella* by Public Health England [16], together with NCTC 5792, serovar Poona (which was not found in EnteroBase by a text search in June, 2020). As shown in Table 2, NCTC 7831 is within HC20_20633 together with 49 other genomes. Strikingly, all but one of them were isolated in Ireland or the United Kingdom, where this strain is recommended for quality control (S9A Fig). (The exceptional genomes was from Poland.) Similar to the HC20 clusters described above, HC20_20633 was isolated over more than a decade (2006-2021). Like HC20_373, many of the older genomes were from Ireland or northern Ireland, as part of the 10 K genomes project [17], but all of those were from veterinary or food safety laboratories where NCTC 7832 was being used for QC of growth of *Salmonella*. Of the entire dataset, 18 are listed as being isolated from food, 14 from sources listed as other, animal, environmental swabs or animal feed, and six from humans. The phylogenetic tree (S9A Fig) resembles that for HC20_373, namely short internal branches and multiple entries from supposedly different sources within several central nodes. The mean pairwise distance within this cluster was 2.6, only slightly greater than for HC20_373 (Table 2). We also searched for serovar Nottingham over all of EnteroBase, and identified a total of only 20 other genomes from six other HC900 clusters, of which HC900_14824 with 10 genomes was the most common. Thus, Nottingham is a rare and genetically polyphyletic serovar. Given the ease with which *S. enterica* can cross-contaminate nutrient media in laboratories and the absence of any confirmation of any of the source attributions of HC20_20633, it is possible that all 49 additional genomes in that cluster represent laboratory cross-contamination. We are therefore hesitant to conclude that any of them were from natural sources.

HC20_5519 (serovar Typhimurium) includes the laboratory strain SL1334, and contained 46 genomes in EnteroBase in June, 2021, with dates of origin ranging from 2002-2018. 31 genomes were accompanied with metadata on their source that were compatible with a variant of the laboratory strain, and only four sets of metadata suggested any other possible origin. We conclude that there is also little convincing evidence for the isolation of this strain from nature. The phylogenetic tree (S9B Fig) resembled those of HC20_373 and HC20_20633, and the mean pairwise SNP distances were 4.6.

Reference List

1. Lerminiaux NA, MacKenzie KD, Cameron ADS (2020) Salmonella Pathogenicity Island 1 (SPI-1): The evolution and stabilization of a core genomic Type Three Secretion System. Microorganisms 8. PM:32316180.

2. Hu Q, Coburn B, Deng W, Li Y, Shi X, Lan Q, Wang B, Coombes BK, Finlay BB (2008) *Salmonella enterica* serovar Senftenberg human clinical isolates lacking SPI-1. J Clin Microbiol 46: 1330-1336. PM:18272702.

3. Ochman H, Soncini FC, Solomon F, Groisman EA (1996) Identification of a pathogenicity island required for *Salmonella* survival in host cells. Proc Natl Acad Sci USA 93: 7800-7804. PM:8755556.

4. Luhmann N, Holley G, Achtman M (2021) BlastFrost: fast querying of 100,000s of bacterial genomes in Bifrost graphs. Genome Biol 22: 30. PM:33430919.

5. Ochman H, Groisman EA (1996) Distribution of pathogenicity islands in *Salmonella* spp. Infect Immun 64: 5410-5412. PM:8945597.

6. Jarvik T, Smillie C, Groisman EA, Ochman H (2010) Short-term signatures of evolutionary change in the *Salmonella enterica* serovar Typhimurium 14028 genome. J Bacteriol 192: 560-567. PM:19897643.

7. Hoffmann S, Schmidt C, Walter S, Bender JK, Gerlach RG (2017) Scarless deletion of up to seven methyl-accepting chemotaxis genes with an optimized method highlights key function of CheM in *Salmonella* Typhimurium. PLoS One 12: e0172630. PM:28212413.

8. Wheeler TJ (2009) Large-scale neighbor-joining with NINJA. 375-389. DOI: 10.1007/978-3-642-04241-6_31

9. Zhou Z, Alikhan N-F, Mohamed K, Fan Y, Agama Study Group, Achtman M (2020) The EnteroBase user's guide, with case studies on *Salmonella* transmissions, *Yersinia pestis* phylogeny, and *Escherichia* core genomic diversity. Genome Res 30: 138-152. PM:31809257.

10. Public Health Agency of Canada (01/10/2020). Public Health Notice: Outbreak of Salmonella infections linked to red onions imported from the United States. https://www.canada.ca/en/public-health/services/public-health-notices/2020/outbreak-salmonella-infections-under-investigation.html.

11. Centers for Disease Control and Prevention (08/10/2020). Outbreak of *Salmonella* Newport infections linked to onions. Final Report. https://www.cdc.gov/salmonella/newport-07-20/index.html.

12. Centers for Disease Control and Prevention (19/10/2017). Multistate outbreaks of human Salmonellosis infections linked to live poultry in backyard flocks, 2017 (Final Update). http://www.outbreakdatabase.com/reports/2017_Backyard_flocks,_final_update_10_19_2017.pdf.

13. Jones G, Pardos de la Gandaro M, Herrera-Leon L, Herrera-Leon S, Varela Martinez C, Hureaux-Roy R, Abdallah Y, Nisavanh A, Fabre L, Renaudat C, Mossong J, Mattheus W, Huard C, Le Borgne C, de Valk H, Weill F-X, Jourdan-Da Silva N (2019) Outbreak of *Salmonella enterica* serotype Poona in infants linked to persistent *Salmonella* contamination in an infant formula manufacturing facility, France, August 2018 to February 2019. Euro Surveill 24: 13. PM:30940315.

14. Ludlam GB, Taylor J, Douglas SH (1953) A new *Salmonella* type of human origin; Salm. Nottingham. Mon Bull Minist Health Public Health Lab Serv 12: 29-30. PM:13025360.

15. NCTC (2021). NCTC 7832: Bacteria Collection: *Salmonella enterica* subsp. enterica serotype Nottingham. https://www.phe-culturecollections.org.uk/products/bacteria/detail.jsp?refId=NCTC+7832&collection=nctc.

16. Public Health England (2019). Detection of *Salmonella* species. National Infection Service, Food, Water & Environmental Microbiology Standard Method FNES16 (F13): version 4. https://assets.publishing.service.gov.uk/ 1-28 https://assets.publishing.service.gov.uk/government/uploads/system/uploads/attachment_data/file/768793/detection_of_salmonella_species.pdf.

17. Achtman M, Zhou Z, Alikhan N-F, Tyne W, Parkhill J, Cormican M, Chiou CS, Torpdahl M, Litrup E, Prendergast DM, Moore JE, Strain S, Kornschober C, Meinersmann R, Uesbeck A, Weill FX, Coffey A, Andrews-Polymenis H, Curtiss 3rd R, Fanning S (2020) Genomic diversity of *Salmonella enterica* -The UoWUCC 10K genomes project. Wellcome Open Res 5: 223. PM:33614977.
