# Supplementary material for: Genomic population structure associated with repeated escape of Salmonella enterica ATCC14028s from the laboratory into nature
Source: PLoS Genet. 2021 Sep 27;17(9):e1009820. doi: 10.1371/journal.pgen.1009820 (PMC8496778; doi:10.1371/journal.pgen.1009820)
Supplement: S2 Table — (DOCX) [file pgen.1009820.s003.docx]

S2 Table. Dated depositions of ATCC14028s derivatives in muliple laboratories, including node assignments and distinctive SNPs.

| EnteroBase metadata | Index (Node) | 1 (F2) | 2 (A2) | 3 (D1) | 4 (D2) | 5 (D3) | 6 (D2) |
| --- | --- | --- | --- | --- | --- | --- | --- |
|  | Strain name | ATCC 14028s | CIP104115 | NCTC12023 | NCTC12023 Holden | NCTC12023 NalR Hensel | NCTC12023 Gerlach |
|  | Lab Source | University of Arizona | Institut Pasteur | NCTC (Colindale) | David Holden | Michael Hensel | Roman Gerlach |
|  | Barcode | SAL_EA9729AA | SAL_FB2028AA | SAL_FB4645AA | SAL_GB2911AA | SAL_GB2910AA | SAL_VA5605AA |
|  | Year deposited | 1960/1961 | 1994 | 1987 | 1995 | 1996 | 2003 |
|  | Accession | CP001363.1 | ERR4993881 | ERR1140974 | ERR5330449 | ERR5330451 | SRR5062192 |
|  | Clade | F | A | A,C,D^1^ | D | D | D |
| SNPs (Node) | 550816  *gsk* (A2) | A | **G** | A | A | A | A |
|  | 2221909  *rfbJ* (A2) | . | **-C** | . | . | - | . |
|  | 3213731  intergenic (A1/C1) | A | A | **A:11 C:42**^1^ | **C** | **C** | **C** |
|  | 609837  *fimH* (C1/D1) | A | A | **A:82 T:28**^1^ | **T** | **T** | **T** |
|  | 2835849  *nadB* (C1/D1) | A | A | **A:65 T:26**^1^ | **T** | **T** | **T** |
|  | 3086350  *rpoS* (D2) | A | A | A | **T** | **T** | **T** |
|  | 2427641  *gyrA* (D3) | T | T | T | T | **C (**D87G) | T |
|  | 1723496  STM14_1964 (F1) | **T** | C | C | C | C | C |
|  | 2480438  *nuoL* (F1) | **A** | G | G | G | G | G |
|  | 4316301  *cytR* (F1) | **T** | A | A | A | A | A |
|  | 2917346  STM14_3323 (F2) | **A** | C | C | C | C | C |

NOTE: SNP positions are according to genome 1 (F2; Accession CP001363). Changes from the putative ancestor in A1 are indicated in Bold. ^1^Genome 3 (NCTC12023) is heterozygous for 3 SNPs that are attributed to nodes C1 and D1.
