# Supplementary material for: Genomic population structure associated with repeated escape of Salmonella enterica ATCC14028s from the laboratory into nature
Source: PLoS Genet. 2021 Sep 27;17(9):e1009820. doi: 10.1371/journal.pgen.1009820 (PMC8496778; doi:10.1371/journal.pgen.1009820)
Supplement: S6 Table — (DOCX) [file pgen.1009820.s007.docx]

S6 Table. Summary of larger InDels observed in HC20_373 genomes.

| Category | Description | # genomes | # distinct inDels |
| --- | --- | --- | --- |
| Lab mutation | *cat:ΔcsrA* | 2 | 1 |
|  | *cat:ΔrelA* | 3 | 1 |
|  | *cat:ΔyebR* | 1 | 1 |
|  | *tet* | 1 | 1 |
|  | Photorhabdus Luciferase | 1 | 1 |
|  | *Tn*10:large deletion | 1 | 1 |
|  | Tn3 lacZ | 1 | 1 |
|  | Tn5:ΔclpS | 1 | 1 |
|  | Tn5:ΔcsrA | 1 | 1 |
|  | Tn5:Δftn | 1 | 1 |
|  | Tn5:Δhns | 12 | 1 |
|  | Tn5:ΔphoN | 1 | 1 |
|  | Tn5:ΔxseA | 7 | 1 |
|  | **Total** | **33** | **13** |
|  |  |  |  |
| Deletions | Δ*fli* cluster | 1 | 1 |
|  | Δgifsy-1 prophage | 4 | 2 |
|  | Δgifsy-3 prophage | 1 | 1 |
|  | Δglycosyltransferase | 2 | 2 |
|  | ΔpSV IncFII(S) | 6 | 1 |
|  | Δ*rfb* cluster | 2 | 2 |
|  | Δ*sseJ* | 1 | 1 |
|  | ΔSPI-1 | 5 | 5 |
|  | ΔSPI-2 | 3 | 3 |
|  | ΔSPI-3 | 1 | 1 |
|  | ΔSPI-4 | 1 | 1 |
|  | ΔSPI-5 | 1 | 1 |
|  | ΔSPI-11 | 1 | 1 |
|  | ΔSPI-12 | 1 | 1 |
|  | **Total** | **30** | **23** |
|  |  |  |  |
| Plasmids | plasmid | 6 | 6 |
|  | ColRNAI plasmid | 1 | 1 |
|  | IncI1-I plasmid | 9 | 9 |
|  | IncI2 plasmid | 3 | 3 |
|  | IncP-1 plasmid | 2 | 1 |
|  | **Total** | **21** | **20** |
|  |  |  |  |
| Prophages | epsilon15-like prophage | 1 | 1 |
|  | lambda-like prophage | 4 | 3 |
|  | P22-like prophage | 4 | 2 |
|  | ST104-like prophage | 5 | 1 |
|  | **Total** | **14** | **7** |
|  | **Grand total** | **98** | **63** |
