# Supplementary material for: Genomic population structure associated with repeated escape of Salmonella enterica ATCC14028s from the laboratory into nature
Source: PLoS Genet. 2021 Sep 27;17(9):e1009820. doi: 10.1371/journal.pgen.1009820 (PMC8496778; doi:10.1371/journal.pgen.1009820)
Supplement: S7 Table — (DOCX) [file pgen.1009820.s008.docx]

S7 Table. Molecular clock rates in other *S. enterica* clades

| Serovar | HierCC Cluster | Clock rate type | Median (95% CI) | Citation |
| --- | --- | --- | --- | --- |
| Agona outbreaks | HC100_29 | Relaxed GMRF | 9.3 × 10^-8^ (0.6 - 1.3 ×10^-7^) | (Zhou *et al.* 2013) |
| Agona outbreaks | HC100_29 | Strict constant | 1.3 × 10^-7^ (1.1 - 1.7 ×10^-7^) | (Zhou *et al.* 2013) |
| Paratyphi A | HC900_587 | Exponential skyline | 2 × 10^-7^ (1.6 – 2.4 ×10^-7^) | (Zhou *et al.* 2014) |
| Typhi H58 | HC20_202 | Relaxed skyline | 1.8 × 10^-7^ (1.2 – 2.6 ×10^-7^) | (Duchêne *et al.* 2016) |
| Kentucky ST198 | HC900_528 | Relaxed constant | 4.8 × 10^-7^ (3.8 – 5.3 ×10^-7^) | (Hawkey *et al.* 2019) |
| Para C Lineage | HC2000_1272 | Relaxed constant | 1.5 × 10^-7^ (0.7 – 2.5 ×10^-7^) | (Zhou *et al.* 2018) |
| Paratyphi C | HC400_4381 | Strict constant | 7.9 × 10^-8^ (0.5 - 1.3 ×10^-7^) | (Zhou *et al.* 2018) |

Reference List

Duchêne S, Holt KE, Weill F-X, Le Hello S, Hawkey J, Edwards DJ, Fourment M, Holmes EC. 2016. Genome-scale rates of evolutionary change in bacteria. *Microbial Genomics* **2**: e000094.

Hawkey J, Le Hello S, Doublet B, Granier SA, Hendriksen RS, Fricke WF, Ceyssens P-J, Gomart C, Billman-Jacobe H, Holt KE, Weill F-X. Global phylogenomics of multidrug-resistant *Salmonella enterica* serotype Kentucky ST198. 2019. *Microb Genom* **5:**  e000269.

Zhou Z, Lundstrøm I, Tran-Dien A, Duchêne S, Alikhan N-F, Sergeant MJ, Langridge G, Fokatis AK, Nair S, Stenøien HK, et al. 2018. Pan-genome analysis of ancient and modern *Salmonella enterica* demonstrates genomic stability of the invasive Para C Lineage for millennia. *Curr Biol* **28**: 2420-2428.

Zhou Z, McCann A, Litrup E, Murphy R, Cormican M, Fanning S, Brown D, Guttman DS, Brisse S, Achtman M. 2013. Neutral genomic microevolution of a recently emerged pathogen, *Salmonella enterica* serovar Agona. *PLoS Genet* **9**: e1003471.

Zhou Z, McCann A, Weill F-X, Blin C, Nair S, Wain J, Dougan G, Achtman M. 2014. Transient Darwinian selection in *Salmonella enterica* serovar Paratyphi A during 450 years of global spread of enteric fever. *Proc Natl Acad Sci U S A* **111**: 12199-12204.
