# Supplementary material for: Genomic population structure associated with repeated escape of Salmonella enterica ATCC14028s from the laboratory into nature
Source: PLoS Genet. 2021 Sep 27;17(9):e1009820. doi: 10.1371/journal.pgen.1009820 (PMC8496778; doi:10.1371/journal.pgen.1009820)
Supplement: S1 Fig — This figure is an alternative representation of the data summarized in Fig 5A. (PDF) [file pgen.1009820.s009.pdf]

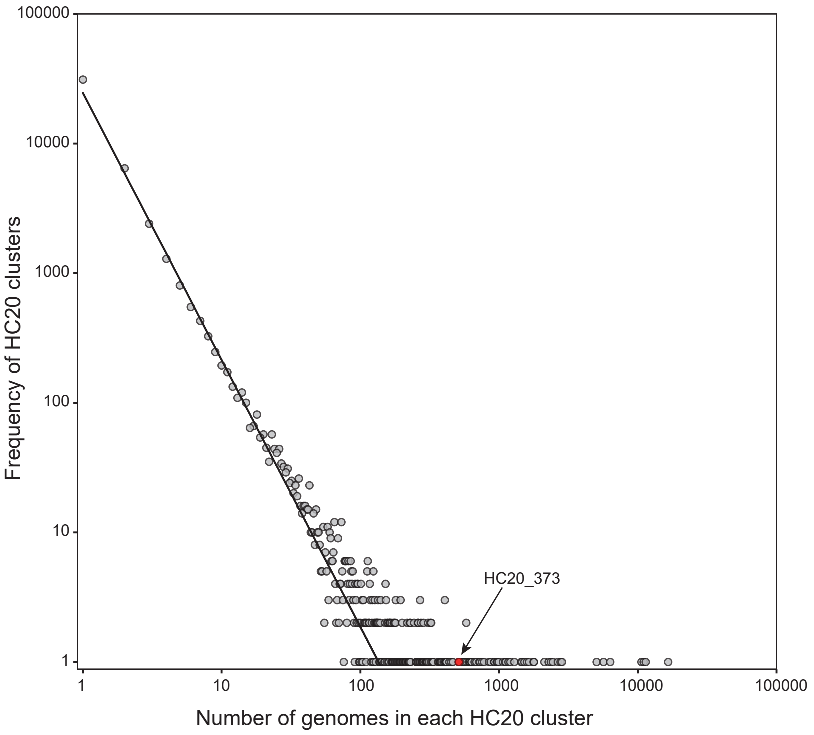

S1 Fig. Log-log plot of frequency of *Salmonella* HC20 clusters by numbers of genomes in each cluster. This figure is an alternative representation of the data summarized in Fig 5A.
