# Supplementary material for: Genomic population structure associated with repeated escape of Salmonella enterica ATCC14028s from the laboratory into nature
Source: PLoS Genet. 2021 Sep 27;17(9):e1009820. doi: 10.1371/journal.pgen.1009820 (PMC8496778; doi:10.1371/journal.pgen.1009820)
Supplement: S2 Fig — (PPTX) [file pgen.1009820.s010.pptx]

## Slide 1
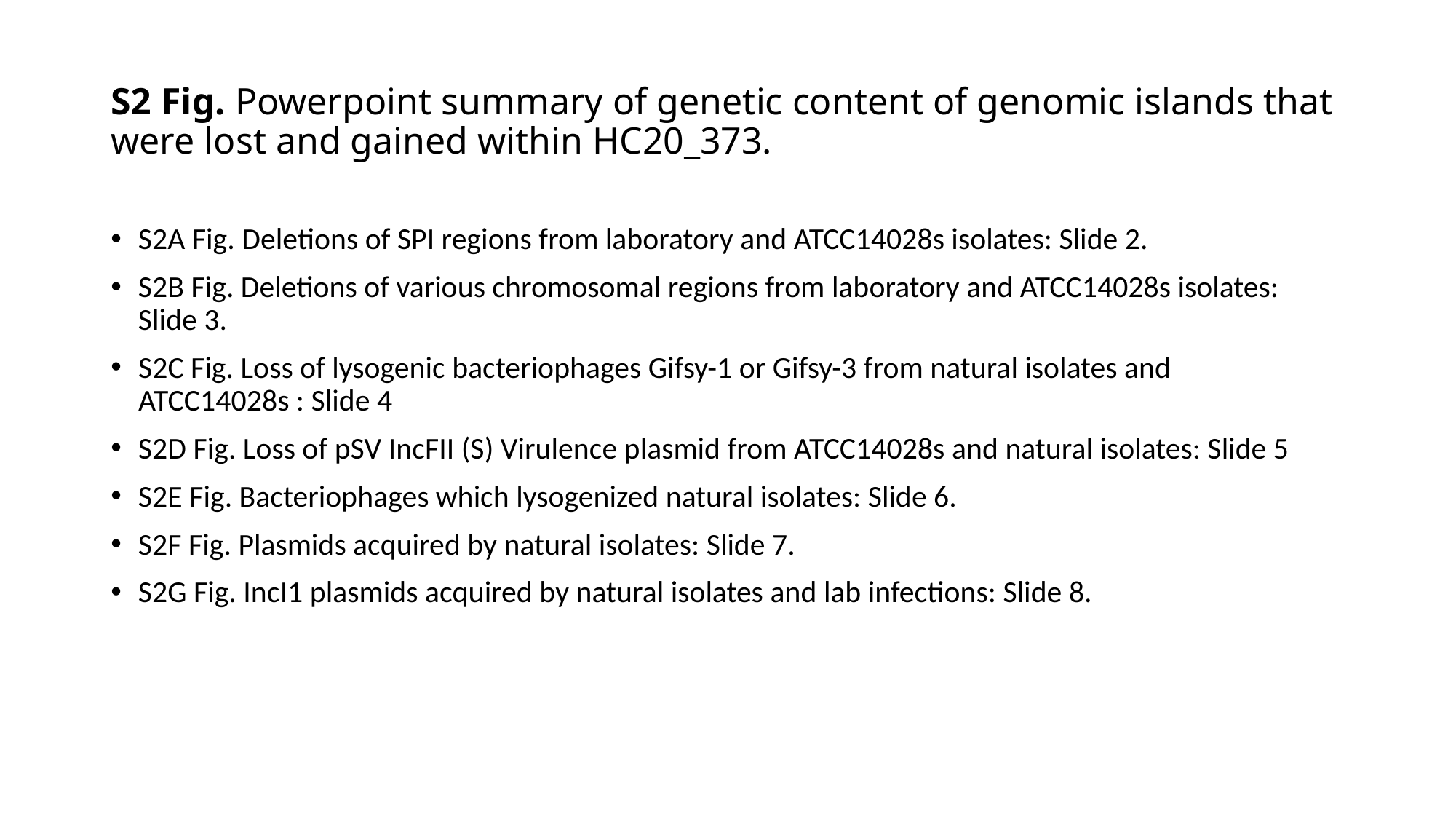

# S2 Fig. Powerpoint summary of genetic content of genomic islands that were lost and gained within HC20_373.
S2A Fig. Deletions of SPI regions from laboratory and ATCC14028s isolates: Slide 2.
S2B Fig. Deletions of various chromosomal regions from laboratory and ATCC14028s isolates: Slide 3.
S2C Fig. Loss of lysogenic bacteriophages Gifsy-1 or Gifsy-3 from natural isolates and ATCC14028s : Slide 4
S2D Fig. Loss of pSV IncFII (S) Virulence plasmid from ATCC14028s and natural isolates: Slide 5
S2E Fig. Bacteriophages which lysogenized natural isolates: Slide 6.
S2F Fig. Plasmids acquired by natural isolates: Slide 7.
S2G Fig. IncI1 plasmids acquired by natural isolates and lab infections: Slide 8.

## Slide 2
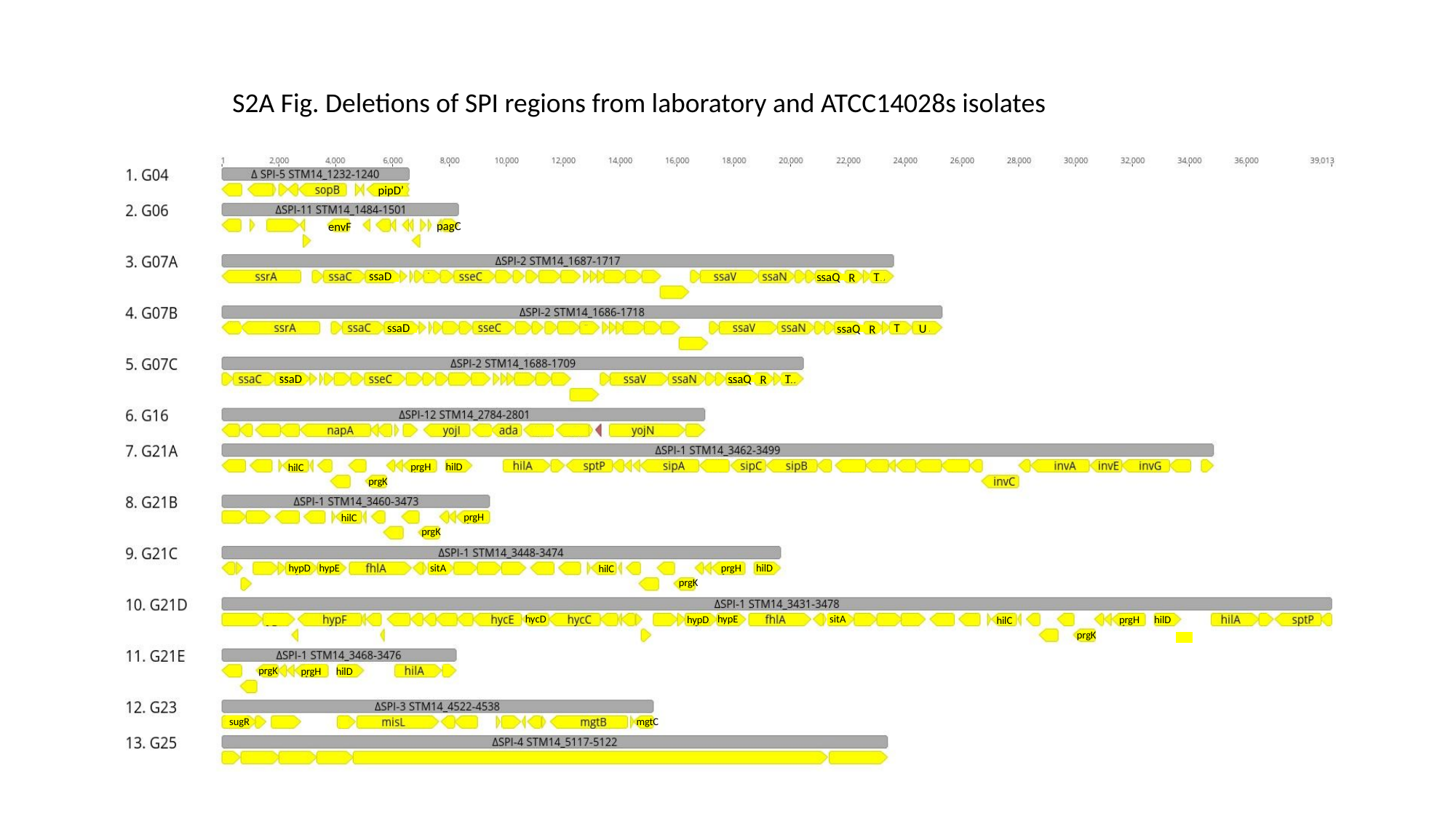

S2A Fig. Deletions of SPI regions from laboratory and ATCC14028s isolates
pipD’
pagC
envF
ssaD
 T
ssaQ
 R
ssaD
 T
ssaQ
 R
 U
 T
ssaQ
 R
ssaD
prgH
hilD
hilC
prgK
prgH
hilC
prgK
hypE
prgH
hilD
hypD
sitA
hilC
prgK
hycD
hypE
sitA
hypD
prgH
hilD
hilC
prgK
prgK
prgH
hilD
mgtC
sugR

## Slide 3
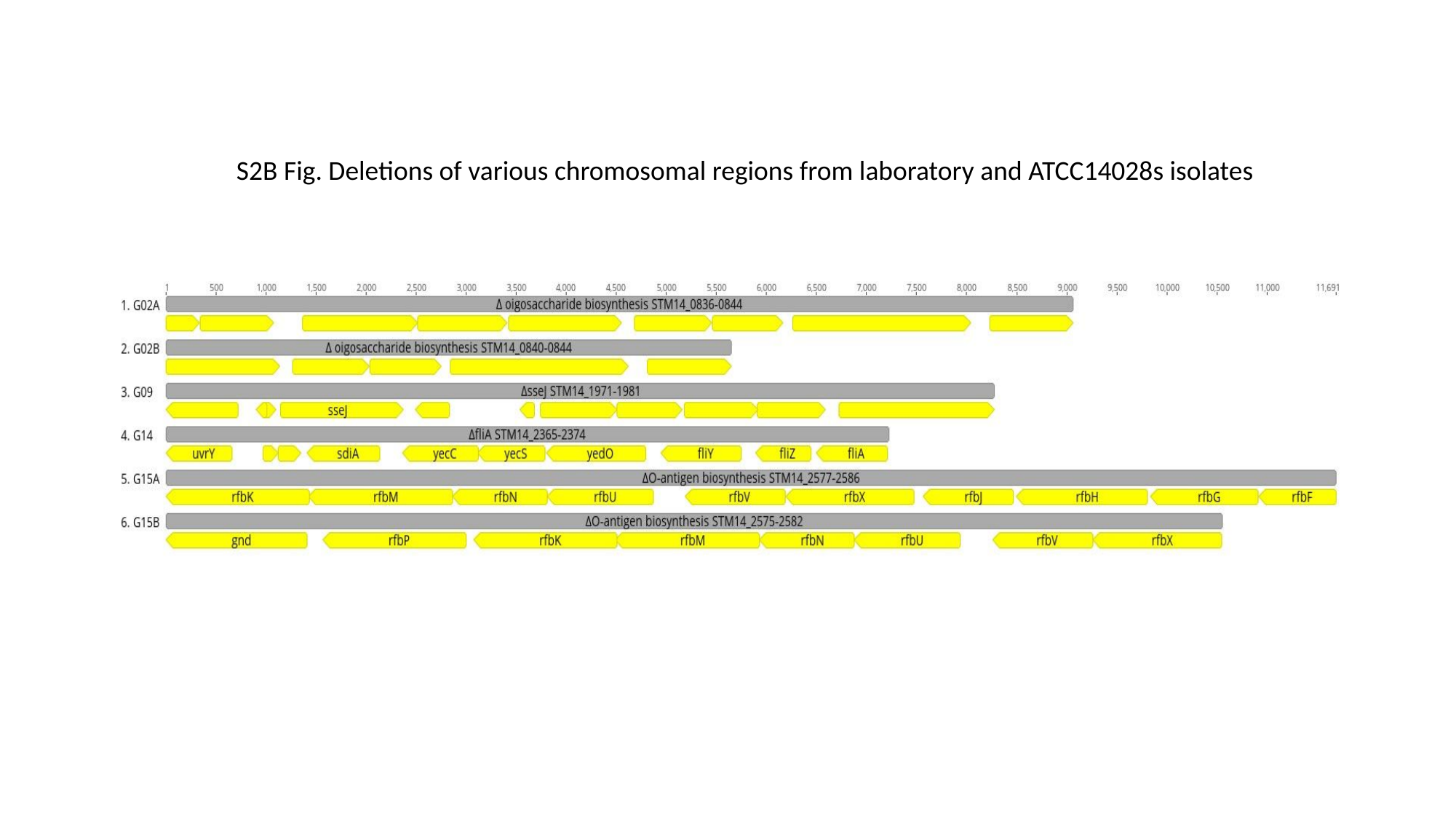

S2B Fig. Deletions of various chromosomal regions from laboratory and ATCC14028s isolates

## Slide 4
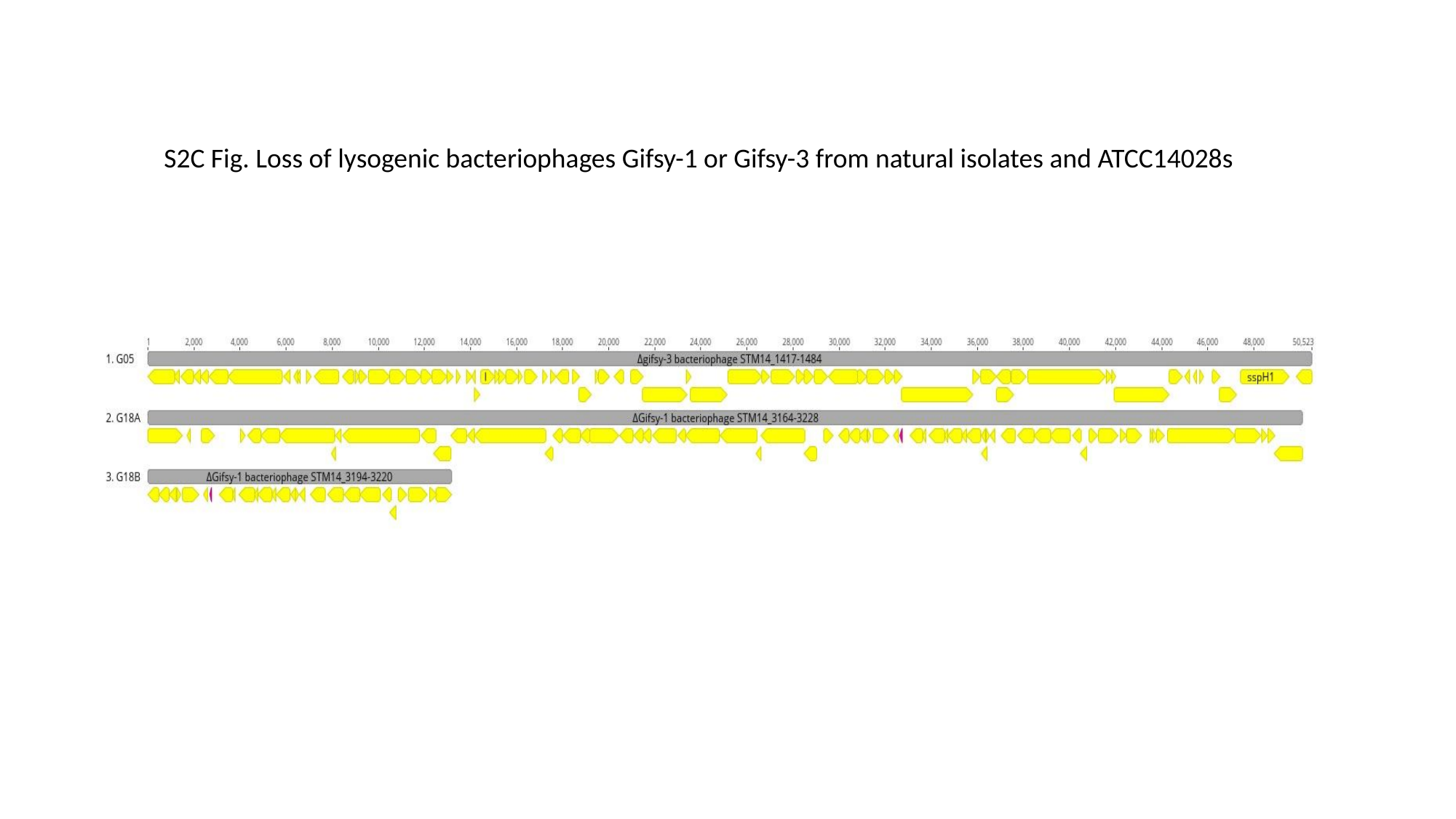

S2C Fig. Loss of lysogenic bacteriophages Gifsy-1 or Gifsy-3 from natural isolates and ATCC14028s

## Slide 5
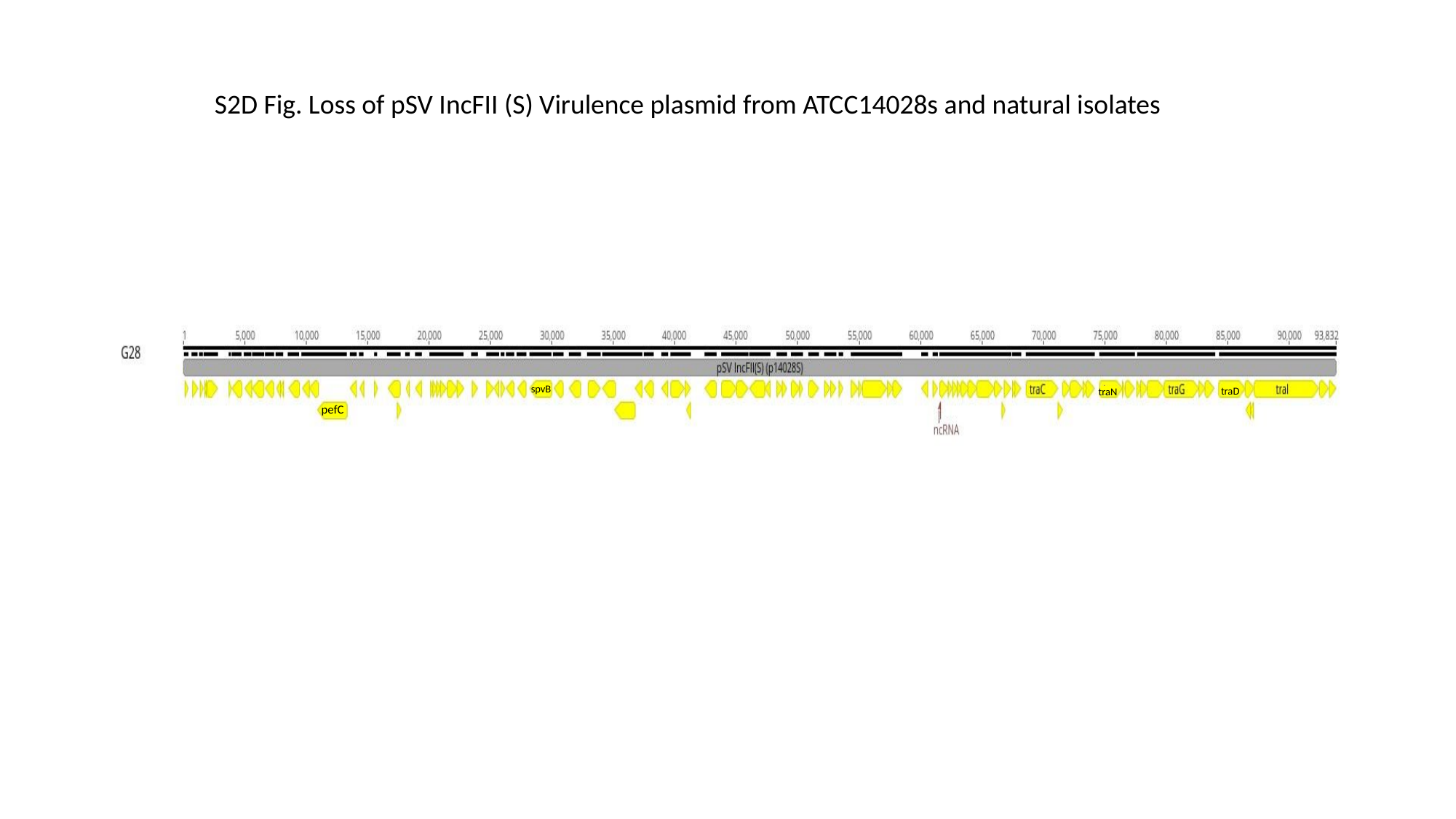

S2D Fig. Loss of pSV IncFII (S) Virulence plasmid from ATCC14028s and natural isolates
spvB
traD
traN
pefC

## Slide 6
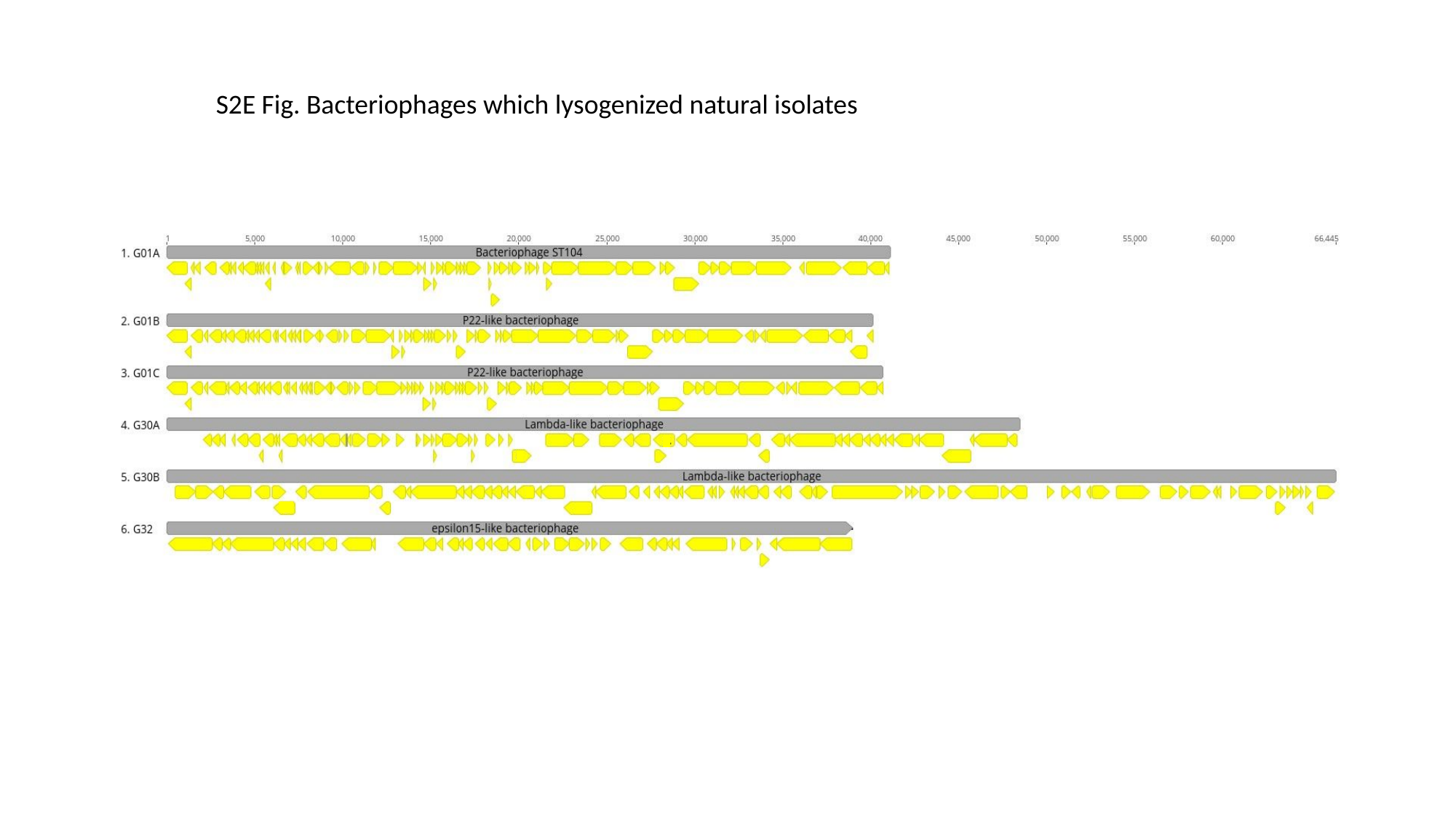

S2E Fig. Bacteriophages which lysogenized natural isolates

## Slide 7
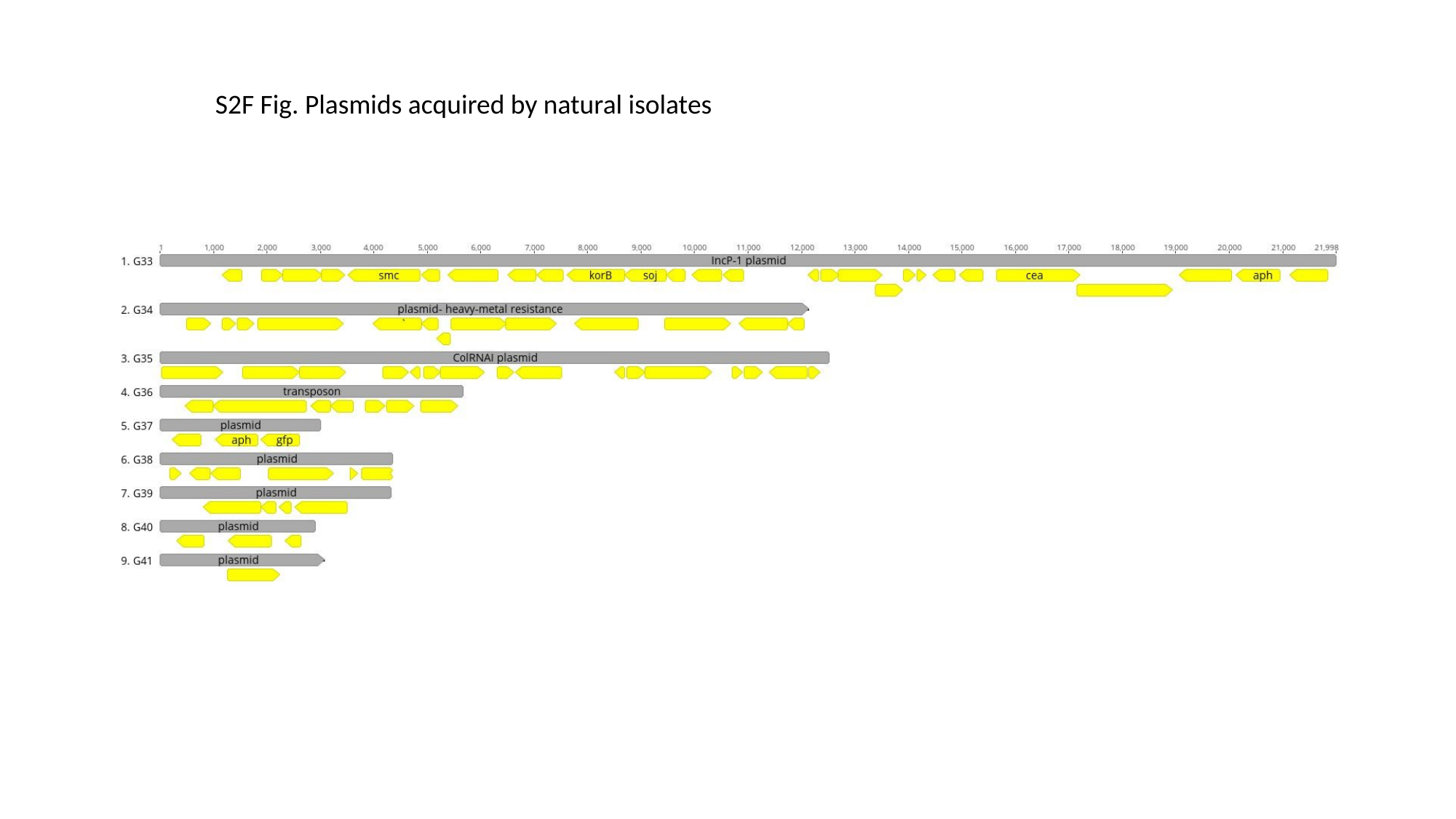

S2F Fig. Plasmids acquired by natural isolates

## Slide 8
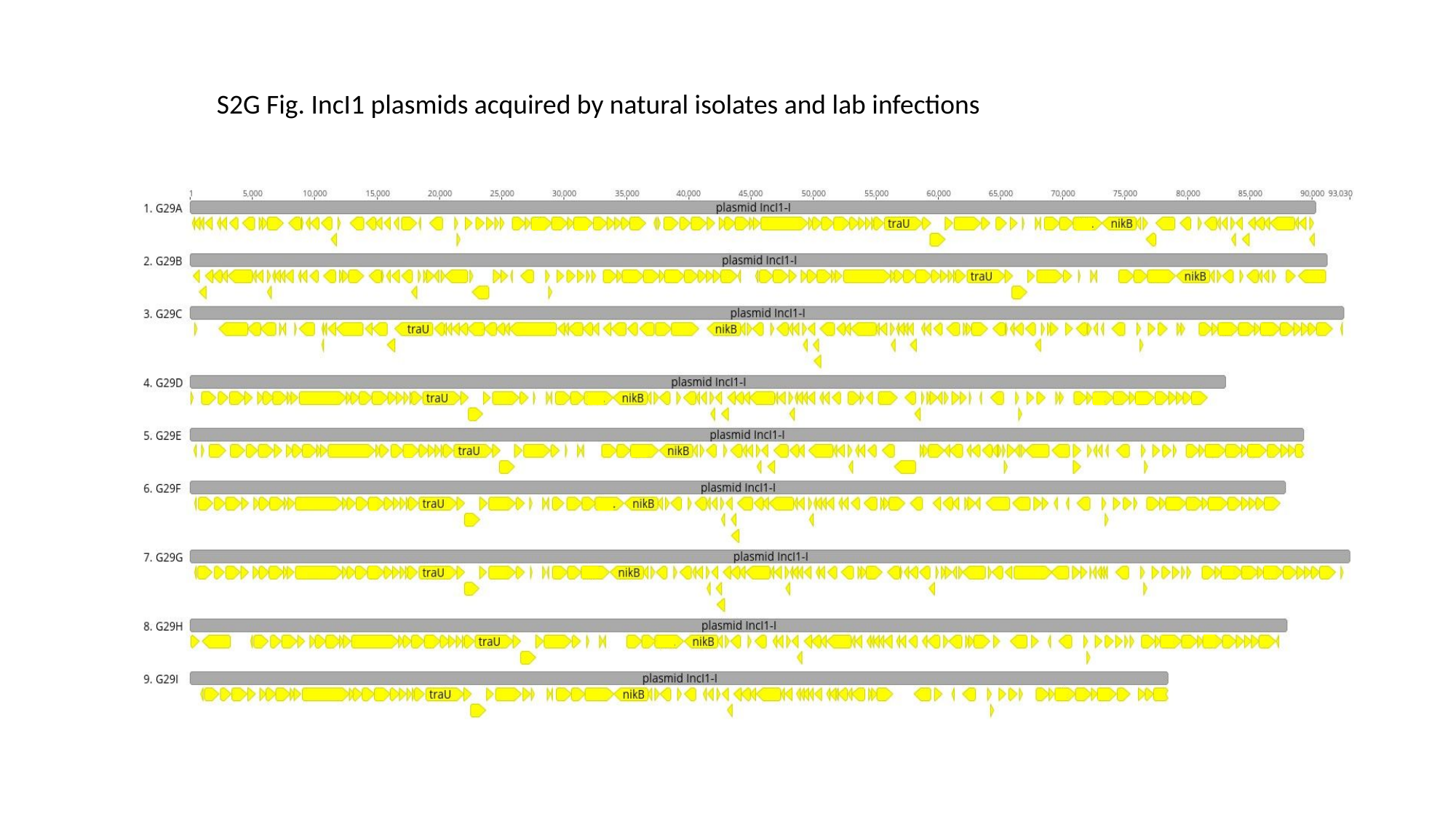

S2G Fig. IncI1 plasmids acquired by natural isolates and lab infections
