# Supplementary material for: Genomic population structure associated with repeated escape of Salmonella enterica ATCC14028s from the laboratory into nature
Source: PLoS Genet. 2021 Sep 27;17(9):e1009820. doi: 10.1371/journal.pgen.1009820 (PMC8496778; doi:10.1371/journal.pgen.1009820)

S3 Fig. Bayesian BEAST [40] temporal dating of ATCC14028s and its natural derivatives. As Fig 6 except that EnteroBase genome barcodes are shown in miniscule fonts at the right of the tree.

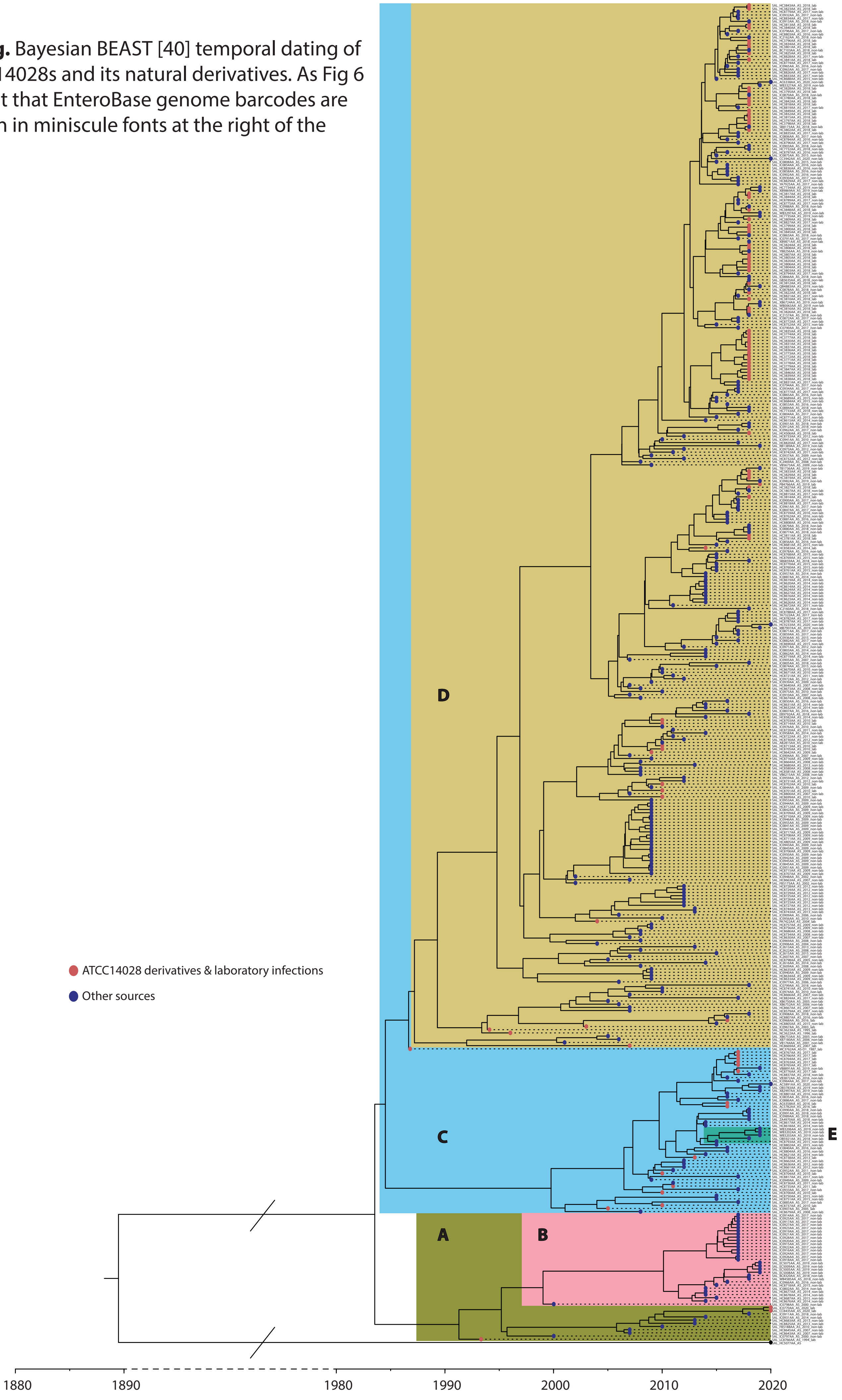

Supplement: S3 Fig — As Fig 6 except that EnteroBase genome barcodes are shown in miniscule fonts at the right of the tree. (PDF) [file pgen.1009820.s011.pdf]
