# Supplementary material for: Genomic population structure associated with repeated escape of Salmonella enterica ATCC14028s from the laboratory into nature
Source: PLoS Genet. 2021 Sep 27;17(9):e1009820. doi: 10.1371/journal.pgen.1009820 (PMC8496778; doi:10.1371/journal.pgen.1009820)
Supplement: S5 Fig — Ninja NJ visualization of allelic differences in the 3002 core genes of the cgMLST Salmonella scheme with GrapeTree for genomes within A) HC20_122 and B) HC20_125. Further information on these HC20 clusters is summarized in Tables 2, 4 and 5, and an interactive version of both trees can be accessed at https://enterobase.warwick.ac.uk/ms_tree?tree_id=45447 and https://enterobase.warwick.ac.uk/ms_tree?tree_id=45450. (PDF) [file pgen.1009820.s013.pdf]

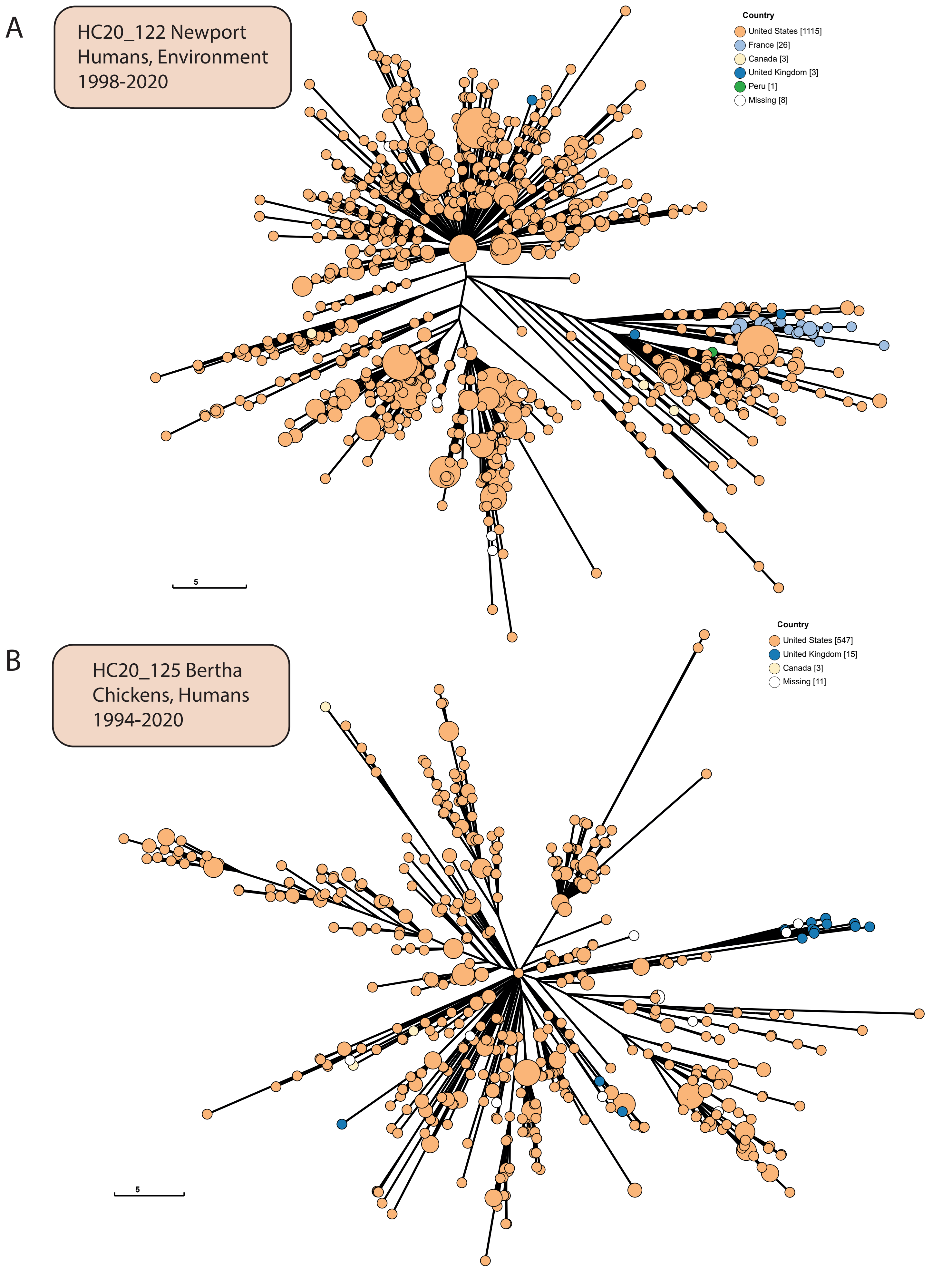

S5 Fig. Ninja NJ [visualization of allelic differences in the 3002 core genes of the cgMLST *Salmonella* scheme with Grape-Tree for genomes within A) HC20_122 and B) HC20_125. Further information on these HC20 clusters is summarized in Tables 2, 4 and 5, and an interactive version of both trees can be accessed at https://enterobase.warwick.ac.uk/ms_tree?tree_id=45447 and https://enterobase.warwick.ac.uk/ms_tree?tree_id=45450.
