# Supplementary material for: Genomic population structure associated with repeated escape of Salmonella enterica ATCC14028s from the laboratory into nature
Source: PLoS Genet. 2021 Sep 27;17(9):e1009820. doi: 10.1371/journal.pgen.1009820 (PMC8496778; doi:10.1371/journal.pgen.1009820)
Supplement: Attachment — Submitted filename: ResponseToReviewers.pdf [file pgen.1009820.s020.pdf]

Reviewer #1: The manuscript is significantly improved with regard to writing. However, it still "basically says that horizontal gene transfer occurs in the wild and that a lab strain cannot survive in the wild. These ideas are already well-established." The authors "agree that the idea that HGT happens in Nature has been widely accepted" but state that "the temporal dynamics of this process have been adequately defined". However, the details of the length of survival of escaped organisms are likely to vary depending on the biology of the organism and the micro/macro environments into which they were released, indicating that they belong in specialty journals unless they reveal larger truths.

Re the second point, that it is well established that lab strains cannot survive in the wild, instead of addressing the criticism, the authors quibble over the meaning of "survival" versus "persistence". They write, "The claim that it is well established that a lab strain cannot survive in the wild is refuted by this paper. ATCC14028s survives quite well… What is being addressed here is whether it (ATCC14028s) will persist". Nevertheless, the manuscript concludes that "the release of ATCC14028s in the environment is not accompanied by long-term persistence and additional microevolution", confirming that there is no new idea here.

Response: There is no way it would be possible to convince this reviewer that this manuscript is interesting to a broad audience. The criticisms are about points that are properly decided by the editors, rather by a reviewer.

Reviewer #4: It remains my opinion that the authors have not unequivocally demonstrated that the data indicates repeated transient escape of a laboratory strain. It is also my opinion that the data suggests that this may happen but great caution with this conclusion is warranted. This is at the heart of the problem I have with the manuscript in its current form: it is written with an unequivocal conclusion that can in fact only ever be equivocal. The title is wholly inappropriate for the level of certainty, and the text also presents this conclusion in a similar tone.

The title has been modified and the text presents the conclusion in a similar tone in all the lines requested by the editor, as indicated in the cover letter. This should satisfy the primary criticisms raised by this reviewer. The remaining comments by this reviewer are predominantly stylistic and/or minor.
