# Supplementary material for: Genomic population structure associated with repeated escape of Salmonella enterica ATCC14028s from the laboratory into nature
Source: PLoS Genet. 2021 Sep 27;17(9):e1009820. doi: 10.1371/journal.pgen.1009820 (PMC8496778; doi:10.1371/journal.pgen.1009820)
Supplement: Attachment — Submitted filename: Reviewers comments.docx [file pgen.1009820.s018.docx]

# Repeated transient escape of a laboratory strain of *Salmonella enterica* into nature

This is an interesting study describing an unusual circumstance where a laboratory strain that has been distributed globally has repeatedly escaped into the environment but has a limited life span. A comparison analysis has been undertaken with proposed ‘natural strains’ which although similar do have a genetic gap and can be differentiated. To differentiate laboratory from natural strains is a difficult task I commend the authors who have undertaken multiple in-depth analyses and formed a cross-cutting and global collaboration to achieve the task.

There are a few comments to address, mainly relating to definitions and addition of information for clarification but recommend this is suitable for publication with revision.

## **Major**

## Background?

Line 57 – 129 – there is no heading for this and it was unclear if this was the background or an extended abstract , it starts of as a background but then specifies a designation of a strain name (would expect this in methods as there is no reference) and then give results and conclusions of the study. This may be a requirement of PLOS so ignore this comment if that is what the journal requires in an introduction.

Line 64 - It is unclear why the strain has been ’designated ATCC14028s here to distinguish it from its rough variants’ . Could you please clarify the reason and what is meant by rough variants, see details below. In the literature, it is referred to as both ATCC14028s and ATCC14028 so please add reference to your statement or clarify in our methods your naming if it is from this study or related to a previous study.

1) ATCC14028s is referred to throughout the manuscript even when referring to defined ‘ATCC laboratory strains’ in the data set which would have been purchased from ATCC so there doesn’t appear to be a differentiation.
2) The term ‘rough strains’ in the laboratory are typically used when a strain has lost its LPS and therefore auto agglutinates with sera and are determined as ‘rough’. Not all ATCC/NCTC strains are rough (in fact they may not be sold if they are). Could you please clarify what is meant by a rough variant?

**There are a lack of definitions of terms and in the manuscript that need to be added to the method or results or explanations are added later in the manuscript so not in order for the reader.**

In the methods, could ‘laboratory derived’ and ‘natural isolates’ selected for the study please be defined for the reader (e.g. is this from the meta data source where ‘human’ are considered natural and ‘laboratory strains’ or ‘other’ are laboratory derived?)

Line 181 - 183 – ‘genetically related’ is a generic term and can mean different things and will have different thresholds (e.g international clones, clades within a phylogeny, outbreak clusters). HC20 has been chosen as a threshold but there is no justification as to why. In the methods please defined the threshold as to what is deemed genetically related, please add a reference if previously done, for the threshold or state the justification so that this study can be used as a reference in the future.

Line 199,213 - ‘laboratory infections’ . How have ‘laboratory infections’ been defined, there is nothing in the methods to specify any epidemiology undertaken so assume this is from the metadata? Please add to methods

Line 206 – unclear as to what CP and CK is (people/organisations), please specify or define sources in methods or refer to a table if it is spelt out there.

Line 277 – specified it has been excluded from this publication but it has been included as you have stated information and have a S1 table. Do you mean that the data was excluded from the dataset for the analysis? If so, please specify this in the methods as part of your selection criteria, why was this excluded if interpretation of results are specified?

Line 278-281 – ‘we also learned’, assumed this from this study but there is nothing in the methods to specify any epidemiological investigation or the source of this information. Please add a section in the methods regarding the source of information if not obtained from the meta data.

### Results

Line 132-158 – this whole page is methods

### Summary

Line 493 – ‘technical staff, in whom it has caused multiple severe infections’. The evidence base for this statement is not currently present in this study, if line 199,213 is revised to clarify how your study identified infections and specify any symptoms to state ‘severe’ then that will provide your evidence base.

## **Minor**

**Title** – specifies ‘Salmonella enterica’ into nature, suggest the title is updated to specify S. Typhimurium since this was known as the start of the study. (just a suggestion)

Line 46 - please clarify what is meant by ‘laboratory evolution’? Control strains are often used a positive controls for a test or experiments but how can a strain be used to look at the evolution of a laboratory?

Line 94 – grammar, conclusions were or conclusion was, which do you mean?

Line 105 – please clarify what you mean by ATCC14028s-like? What is meant by like? Since this the introduction with a background on ATCC14028, why is ATCC14028s referred to in the background, since this is a new definition in your study?

Line 122-129 – Results don’t typically do into an introduction. Please clarify what is meant by ‘human communications’ spreading a clade. Do you mean ‘contamination’?

Line 118 – Please specify time period/year for the referred reference as ‘currently’ will change as the manuscript gets older.

### Results

Line 133 – need to add somewhere regarding metadata since the genomes will support the biology but the metadata will support geographical sources.

Line 174,416,458 – please clarify what is meant by ‘sibs’ for the reader, and throughout the manuscript. Do you mean sibling (not a bacterial term) or susceptible indigenous bacteria (SIBs) (doesn’t fit in with the context of this manuscript).

Line 177, 427 – Please change term from ‘accidents’ to ‘incidents’ throughout the manuscript as appropriate (unless you are referred to a specific accident) , there doesn’t always have to be an ‘accident’ for contamination, or a laboratory acquired infection to occur.

Line 184 – states that a NJ tree was drawn, couldn’t see this in the methods (only the ML tree), could this please be added to the methods.

Line 229 – please specify year of isolate rather than ‘early isolate’ (or if you don’t mean year, please clarify ‘early’)

### Discussion

Line 388 – please spell out SNV in the first instance

422 – please clarify what you mean ‘by post’

Line 442 – please use alternative phrase to shed (used for when a patient shed/excretes a pathogen, so not appropriate in the context of a laboratory). Contaminated/ escaped ?

### Summary

Line 490 – ‘model organism for laboratory infections’ – please clarify for reader, do you mean laboratory acquired infections or laboratory experiments such as infection models?
