# Supplementary material for: Genomic population structure associated with repeated escape of Salmonella enterica ATCC14028s from the laboratory into nature
Source: PLoS Genet. 2021 Sep 27;17(9):e1009820. doi: 10.1371/journal.pgen.1009820 (PMC8496778; doi:10.1371/journal.pgen.1009820)
Supplement: Attachment — Submitted filename: Response to ReviewersV3.docx [file pgen.1009820.s019.docx]

Response to Reviewers:

Reviewer 1:

We are very pleased for the kind words by this reviewer about previous publications. We agree that people who work on *S. enterica* and lab safety microbiologists will be interested.

We agree that the idea that HGT happens in Nature has been widely accepted. It is not as clear to us that the temporal dynamics of this process have been adequately defined, and this is one of the first attempts to elucidate the details of the length of survival of escaped bacteria in nature.

The claim that it is well established that a lab strain cannot survive in the wild is refuted by this paper. ATCC14028s survives quite well, and seems to be happy in the Salinas river and in soil and in vegetables. It continues to infect humans over decades in Ireland and Taiwan. It is clearly surviving. What is being addressed here is whether it will persist, which is a different topic, and one which is poorly understood.

The authors of this paper come from the fields of food safety, virus infections and bacterial population genetics. Each of us has learned an enormous amount about developments in the other disciplines during the writing of this manuscript and our interactions. And although each of us knew of examples of pathogens escaping from laboratories in our areas, none of us were aware of the variety of such events that are summarised here. And none of us were prepared for the dimensions of laboratory escape that are documented in this manuscript. As a result, we disagree with the reviewer about their evaluation of the interest this manuscript will stimulate and its importance to microbiology.

Reviewer 2:

We thank reviewer 2 for the glowing praise.

Minor comments:

1. Why is the proportion of infections of humans different between HC20_373 and other related HC_20 groups? Answer: We don’t know.

This is not for lack of trying. We have been looking at multiple HierCC groups and trying to look for similarities and differences. It is difficult to find general rules. However, reviewer 4 asked for more evidence that HC20_373 has escaped transiently, and as a result the revised manuscript contains multiple additional examples of HC20 clusters and their properties. There are some trends, but we still don’t know why some HC clusters are so specific in location or host.

2. As indicated in the text, founder nodes radiate descendants. A founder is the ancestor of a cluster or clade. Such clusters tend to have fewer descendants at the tips than at the central node, which is thought to be the founder node. These are concepts developed in the original descriptions of population structures in bacterial pathogens. Since this is a common term, we have now included the designation “so-called founder nodes” in the manuscript.
3. tMRCA 20 years after isolation. This topic has plagued M.A. for a long time. It was addressed in citation 49 ( <https://pubmed.ncbi.nlm.nih.gov/30033331/>). Basically there is a crown group that has an MRCA but the root of the lineage is when it split away from other lineages, which is earlier than the tMRCA for the crown group. As it turns out, recalculating the data with a relaxed clock rather than a strict clock for this revision moved the date back to 1971, which is only 11 years after the isolation date.
4. Node C1. This was a typo left over from a previous version with different nomenclature for the nodes. It is A1 as indicated in the figure legend and has now been corrected in the manuscript.
5. The effect of bottlenecks is to reduce diversity because it purifies away low levels of variation. Bottlenecks are also associated with explosions of diversity because multiple SNVs can pass through a bottleneck if they happen to be linked in the founder cell. The reviewer is correct that the evolutionary rate is lower for ATCC4028s than for some natural clusters of *S. enterica*. The evolutionary rate was somewhat higher in this revision because of using a relaxed clock. This revision also provides clock rate estimates for multiple other HC20 clusters, all of which are somewhat higher than HC20_373.
6. More cause -> more likely cause
7. Percentages now explained in the figure legend to Fig. 4. These were the frequencies of the heterozygous SNPs in the completed genome of NCTC 12023.

Reviewer 3:

We are pleased that the reviewer found the manuscript interesting. Somehow line numbers disappeared in the original submission so the numbers used by the reviewer do not correspond directly to the new numbers.

Major points

Line 57-129 (now 63 ff): This is Introduction, and now labelled as such. The strain name CDC 60-6516 is described in citation 1, which is at the end of the sentence. PLoS does not require this form of an Introduction, and the style was determined by the authors.

Line 64 (72 ff): We now explain that rough variants in the sense used in explanation 2) did arise during storage and were inadvertently distributed by the ATCC until they realised that this was the case. We cite citation 1 where this is elaborated. One of those customers was the Institut Pasteur which received a mixed culture of rough and smooth variants but only kept the smooth variant. Subsequent sales by the ATCC were of the smooth variant after re-isolation without a change in designation.

Lack of definition of terms:

p. 8, l. 169 ff now reads. “In order to identify laboratory derivatives of ATCC14028s, we searched EnteroBase for strain names and other metadata containing variants of “ATCC14028”, “60-6516”, “NCTC 12023” or “laboratory evolution”. Manual curation of the metadata and information exchanges with the laboratories responsible for the genomic sequencing led to the assignment of “ATCC14028s derivative” for multiple entries which had initially appeared to be of natural origins, and to the exclusion of two unreliable genomic assemblies from further analysis (Supplemental Table S1). We also identified and sequenced several additional strains from institutional and individual strain collections with a known history of derivation from ATCC14028s and known dates of acquisition, and included them among the ATCC14028s derivatives (Supplemental Table S2). The final dataset consisted of 156 ATCC14028s derivatives (Table 1).”

The suggestions by the reviewer of depending solely on metadata would not have been as thorough as the procedures summarised above. It would be possible to move all this text to Methods if required but would make the manuscript more stilted and less clear. Instead we have modified Methods l. 642 ff to read:

155 genomes were assigned to the category ATCC 14028s derivative as described in Results. We also assigned 17 genomes to laboratory infections due to publications [9,10] (11 genomes), epidemiological investigations in France (3 genomes; see results) and/or based on public metadata (3 genomes). Other genomes were assigned to the other categories used to color-code Fig. 4, and all these assignments were stored in the “14028s” user-defined field within EnteroBase [15].

p. 8 line 181-183. Genetically related. Reviewer 3 requests either a reference for using HC20 as a threshold or a justification. The implication is that this topic has been settled. Unfortunately that is not the case.

As indicated in lines 164-168, epidemiologists are starting to use HC5 or HC10 clusters which occur within a limited time frame to identify single-source food-borne outbreaks. Clearly a strain that has been distributed globally since 1960 does not fit this criterion. But we have no published overview of what to expect. According to the EnteroBase User’s Guide published in 2020 (citation 15), Agama strains form an HC2000 cluster whereas local transmission changes between badger sets correlate with HC10 clusters. However, that paper also describes an HC10 cluster infecting humans in France and Austria, indicating that transmission chains might also involve clusters of this order of magnitude.

This manuscript describes how we defined the cluster size to investigate. The text (l. 199 ff.) now reads:

ATCC14028s must have existed in the environment prior to its isolation in 1960, and its close relatives (sibs) and descendants might conceivably be common among natural isolates today. ATCC14028s has also subsequently escaped from the laboratory on multiple occasion via repeated contamination of sewage systems with feces from the hundreds of people infected with ATCC14028s in 1984 during the bioterrorism attack [6] or in subsequent laboratory accidents [9,11–13]. Natural sibs and descendants of ATCC14028s would be expected to show more genetic diversity than laboratory-derived strains due to the effects of large population sizes, neutral drift and selection in nature. We tested these expectations by examining the genetic diversity of *S. enterica* HC50_147, a higher order hierarchical cluster which includes HC20_373 and other related HC20 clusters.

On Dec 1 2020, HC50_147 included 2098 entries from natural sources (Fig. 2). All other HC20 clusters within HC50_147 differ from HC20_373 by at least 20 distinct alleles. We show below that the time to their most recent ancestor (tMRCA) with HC50_147 was before 1900. Any recent, natural descendants or sibs of ATCC14028s should therefore be restricted to HC20_373.

This does not directly address the request by the reviewer. But it justifies using this cut-off. We also note that other reviewers requested additional information on the genetic diversity of other laboratory strains and their natural relatives as well as on non-lab strains. These are now also included in the manuscript, as described in the responses to the other reviewers.

Line 199, 213 (642 ff.). The basis for the assignments to laboratory infections and other categories in Methods have now been amplified and read:

155 genomes were assigned to the category ATCC 14028s derivative as described in Results. We also assigned 17 genomes to laboratory infections due to publications [9,10] (11 genomes), epidemiological investigations in France (3 genomes; see results) and/or based on public metadata (3 genomes). Other genomes were assigned to the other categories used to color-code Fig. 4, and all these assignments were stored in the “14028s” user-defined field within EnteroBase [15].

Line 184 (240). Now designated C.P. and C.K. and they refer to two of the co-authors.

Line 277 (377). Now reads: excluded from further analysis in this publication

Line 278-281 (379-382). Now reads Of four HC20_373 isolates from human infections in France, enquiries to the responsible epidemiologists revealed that one was from a laboratory infection and two others were from a mixed *Salmonella* infection of a truck driver associated with contamination during the transport of biological waste.

Results Line 132-158 (149-168). The reviewer states that this is all Methods. We disagree. This is a necessary description of HierCC for an audience who has never dealt with EnteroBase. If it moved to Methods, it would be impossible to understand the results without having first read Methods.

Minor points

Title. We thank the reviewer for the suggestion but prefer the current title.

Line 46 (24): now reads experiments on fitness (“laboratory evolution”)

Line 94. Corrected

Line 105. The reviewer does not seem to have read citation 1, which introduced the designation ATCC14028s and also summarised its history as well as describing complete genomes.

Line 122-129 (137-146). 20 years ago M.A. learned from a master of writing manuscripts, John Maynard Smith, that an exciting manuscript started with a question in the first paragraph of the introduction and finished with a summary of what was to be proven in the last paragraph. This advice has served him well and he is so close to retirement that he is not willing to change his style.

Shipping bacteria from one place to another is one form of human communications, as is asking your post-doctoral supervisor for permission to take a sub-culture of an important bacterial strain with you when you leave. In the past, exchanging bacterial strains between laboratories was a very enjoyable form of communication between (human) scientists. As was writing letters.

Line 118. Currently now replaced with a time period.

Line 133 (132). Now reads: However, in early 2021, EnteroBase [15] contained draft genomes and their metadata from more than 280,000 *Salmonella* isolates and their metadata from diverse sources around the globe, including many from environmental sources.

Line 174, 416, 458 (96, 200, 213): sib. According to Wikipedia,

[Sib (anthropology)](https://en.wikipedia.org/wiki/Sib_(anthropology)), a group of people recognized by an individual as kin

I would expect most readers of PLoS Genetics to know what a sib is.

Accident vs incident. An incident has a connotation of criminal behaviour. An accident is something that happens to everybody, but some unfortunates get punished. There is a long history of referring to lab accidents as lab accidents, and I see no reason to change this term in this manuscript.

Line 194. The figure legend in Fig. 2 includes both the terms NJ and Neighbor joining and cites using GrapeTree for creating the Ninja NJ tree.

Line 229. Unfortunately, the date of isolation of this isolate is unknown other than that it was early.

Line 338. Done

Line 422. Done

Line 442 escaped instead of shed

Line 490. Done

Reviewer 4:

We thank the reviewer for concluding this manuscript was technically excellent and well-presented. However, we disagree that the conclusions that HC20_373 has escaped repeatedly is not well supported. The reviewer states correctly that we did not adequately address whether the limited diversity or multiple short chains were atypical. This was due to an attempt to keep the manuscript from being too detailed for a general audience, and because we were concerned that bringing in additional data would make it more difficult to make our points. However, we have shared the same concerns since the beginnings of our investigations, and had already analysed additional comparable populations of natural isolates. We had anticipated bringing out a separate manuscript describing their properties but failed to learn anything exciting from our analyses. Due to the reviewer’s comments, these data are now included in the manuscript and will not be published elsewhere. In an attempt to preserve a semblance of brevity, the trees and detailed descriptions for these analyses are presented as supplemental material.

Major changes in the manuscript:

1. New figures, Fig. 5 and Fig S1, present a detailed overview of the frequency distributions of HC20 clusters by numbers of entries in a log-log histogram (Fig. 5A), and focusing on larger HC20 clusters comparable in size to HC20_373 (Fig. 5B). Previous Figs. 5-6 are now 6-7.
2. A new table, Table 2, present the general properties of 8 larger HC20 clusters which were isolated over extended time periods, two other larger HC20 clusters which have the properties of food-borne outbreaks, as well as the HC20 clusters for 3 lab strains: ATCC14028s, SL1344, and NCTC 7832. These properties include summary data for the mean, median and maximum pairwise non-repetitive SNP distances across each HC20 cluster, as well as a summary of serovar, geography, predominant host and dates of isolation. The corresponding Ninja NJ trees of allelic distances of cgMLST are presented in supplementary Figs. S3-S9. A detailed overview of this new data is presented in supplementary text under the headings Random large HC20 clusters, Outbreaks associated with HC20 clusters, HC20 clusters of laboratory strains, NCTC 7832, and HC20_5519 (lines 52-140). The properties of seven additional large HC20 cluster properties are included in a new Table S4, Former Tables S4-S5 have now been renamed to S5-S6. Former Table S6 has been deleted and Table S7 is the same.
3. Table 4 now contains the GSS data for a comparison of strict and relaxed clocks in 11 HC20 clusters from Table 2. Table 5 contains the corresponding clock rates and tMRCA values for the same clusters. We have rewritten the text for lines 468-511

We had already studied most of these populations prior to submitting the first version of this manuscript, and the new analyses performed now confirmed our conclusions that HC20_373 is different from other laboratory strains and from natural populations.

The reviewer also raises the point that natural isolates do not show signs of genetic manipulation in the laboratory prior to escape. In the original version of this manuscript, we were concerned that there were any natural isolates at all with signs of genetic manipulation. But the reviewer is correct that some manipulated strains should also have escaped, and a few did. The frequency of such escape is a function of how many labs were performing genetic manipulations and also allowing the progeny to escape. This seems to have been only a few, and indeed most of the genomes of lab strains with signs of genetic manipulations were produced by a limited number of scientists.

The reviewer claims that it is implausible that such high numbers of natural infections can be sustained by a strain that is only transiently present outside the laboratory. There is an assumption here of something we never claimed. We did not claim that ATCC14028s was escaping the laboratory and only surviving for days. We also did not claim that all natural isolates of ATCC14028s were isolated from zoonotic infections. Instead numerous isolates of HC20_373 were from water, soil, or plants. And the time-dated tree of HC20_373 spans a range of decades, not days. We argue that the escaped bacteria are transient in nature, but transience can be measured in years and still be transient.

The reviewer criticises our “sweeping statements …presence of phage and plasmids is evidence of having circulated in animal populations, ignoring the possibility that these have been acquired by HGT in the lab”. We have re-examined all mobile elements, including identifying the taxa of the hosts of the mobile elements with the best BLAST hits. This resulted in assigning one former supposed natural isolate from poultry to the category of ATCC14028s derivative because it contained bacteriophage lambda with marker genes for following bacterial infections and the public record indicated that the genome came from veterinary laboratory investigations. The closest BLAST hits for all other phages and plasmids attributed to HGT were in genera distinct from Salmonella or from natural Salmonella serovars that cause infections of humans and domesticated animals. Why does the reviewer invoke mixed cultures in the lab of ATCC14028s with odd taxa from the tree of life containing unknown and undescribed plasmids and bacteriophages as a logical argument? Why would a scientist performing lab evolution of virulence mix their cultures with uncharacterised wild strains of other phyla and undefined bacteriophages? And why were these not found in the known lab strains? This criticism has no scientific or logical basis. We also contacted Eduardo Rocha who has considerable experience on mobile genetic elements in natural strains, and he agreed with our conclusion.

General comments:

1. No line numbers. Now corrected. This reflected an inadvertent mistake during submission of a text which previously contained line number.
2. Difference between animal and human isolates with plasmids and prophages. Table 1 has one column for 159 Human Non-laboratory isolates and separate columns for poultry, swine, cows, Plants or Food, Soil or Rivers, and Animal Feed, Llama or Lamb. The rows contain all the numbers requested by the reviewer.
3. Atypical diversity of HC20_373. Variation in diversity at HC20 level. Other low diversity HC20 clusters. Other clonal groups available from ATCC or used in research labs. Analysis of serovar Nottingham NCTC 7832. Table 2, Fig. 5, Supplemental text answer all these questions and confirm our prior conclusions. Indeed, the suggestion of looking at NCTC 7832 proved particularly revealing. 49/50 genomes of this HC20 cluster are from the UK or Ireland and we unable to find convincing data that excludes lab contamination. In contrast, ATCC 14028s was isolated much more frequently from humans in the UK or Ireland than NCTC 7832 although it is not recommended there for QC.
4. Transposons in lab strains of ATCC14028s but not in natural strains. Why? We don’t know.
5. Repeat of Question 4 with other words. We don’t know. However, we did find standard lab transposons used for recombineering in lab strains (cat, Tn5, Tn10, tet, Tn3, Luciferease; see Table 1).
6. How many labs in the UK culture ATCC14028s? We don’t know. The reviewer seems to be presuming that lab escapees survive only days. Instead we are finding human infections in Ireland and Taiwan with HC20_373 over periods of decades. Multiple labs that do not cultivate ATCC14028s have isolated HC20_373 repeatedly from the Salinas river and plants in California. Two of 1500 distinct outbreaks of human Typhimurium in Denmark were caused by HC20_373. This strain is coming up in isolates from domestic animals in France and England and Germany.
7. Plausibility not addressed. The reviewer would like to see theoretical calculations or models that could quantify the degree with which HC20_373 is escaping the laboratory. At the moment, we don’t even have estimates of how long it can exist in nature, so mathematical modelling seems a bit premature. If the reviewer wished to contact us, we would be enthused about collaborating on a modelling exercise but do not feel competent to do this ourselves.
8. Significance of different frequencies of SPI deletion not addressed. We agree completely. We have reduced this to one sentence and moved the prior text to Supplemental text. We also don’t know what it means but suspect it is random noise.
9. GSS too technical. We agree. It has now been rewritten for a more general audience. The reviewer recommends we delete it totally. We disagree because it indicates there is convincing statistical support for a temporal signal, which was a prerequisite for calculating the evolutionary signals. The substitution clock rate proved to be slower than in natural HC20 clusters, which is now discussed briefly.

Our general response is that we have responded adequately to the general criticisms made by the reviewer, but were not able to address all the questions and critical comments, in part because some of them are inappropriate.

Specific comments

1. Abstract. The sentence now reads: “The isolation history of some of the natural isolates confirms the conclusion that they do not represent recent contamination by the laboratory strain, …”
2. P.13-14 (Now p. 11). Disbelief and absence of data. First paragraph: We deliberately presented epidemiological details for a few cases in France to illustrate what the reviewer is claiming, namely that some apparently natural isolates are lab contamination. The baby is one example of many where our attempts to find possible sources of lab contamination were refuted by the lab scientists and epidemiologists. We now state: “We also corresponded with the individuals who had deposited their short-read sequences in EnteroBase (the ATCC14028s Study Group) in order to identify and exclude misattribution or laboratory cross-contamination among supposed natural isolates.”

Second paragraph: The reviewer is misconstruing our claims. We make no statements whatever about which labs are releasing the bacteria into nature because we have no data on source. Instead, we are simply addressing the chances that the genomes were from contamination in the lab with the lab strain because it had been used in parallel for QC during isolation. However, we now present a comparison of relative frequencies of HC20_373 from humans and other sources in the British Isles in comparison to the recommended QC strain. The comparison is very compelling that HC20_373 infects many more humans than would be expected for laboratory cross-contamination (lines 388-398).

3. P. 21. Rivers and on plants. Rivers (lines 233, 237, 241; 552; Table 1). Plants (line 234, 236, 241: (one strain each from cantaloupe, celery, nuts and corn soy blend). Abstract lines 54-60 now reads: “We present data indicating the ATCC14028s has repeatedly escaped from the laboratory through inadvertent contamination of the environment, infection of technical staff and deliberate bioterrorism. The escapees survive in nature long enough that some acquire mobile genomic elements by horizontal gene transfer, but eventually they go extinct. As a result, even extensive global databases of natural isolates lack closely related isolates whose ancestors diverged from ATCC14028s within the last 100 years.”. The last paragraph of the discussion reads “The data presented here show that HC20_373 has escaped from the laboratory on multiple occasions, contaminated the environment and food, and infected humans and other animals around the globe. Genomes from natural isolates of HC20_373 comprised more than 1% of all Salmonella genomes in EnteroBase, i.e. these bacteria are more frequently isolated than are Salmonella of many rare serovars. It is not possible to confirm that all “natural” isolates were truly natural rather than resulting from laboratory contamination. But the identification of novel plasmids and lysogenic bacteriophages in 9% of natural isolates is a reliable indicator that many or most of them are truly natural. Thus, HC20_373 exists in nature. But it does not seem to continue to transmit indefinitely, and the phylogenetic structure of the natural isolates corresponds to that of repeated extinction events after multiple escapes. **“**

l. 107-109. “This conclusion was also supported by a phylogenetic SNP tree of 29 genomes isolated from laboratory infections with ATCC14028s and natural isolates from lettuce and other food products [10].”

The reviewer may be unaware the *S. enterica* infects plants and colonises the environment. In that event, I would recommend reading citation 14 where the Introduction states: “We note that S. enterica can be isolated from rivers, ponds and drinking water ([Meinersmann et al., 2008](https://wellcomeopenresearch.org/articles/5-223#ref-44); [Uesbeck, 2009](https://wellcomeopenresearch.org/articles/5-223#ref-58); [Walters et al., 2011](https://wellcomeopenresearch.org/articles/5-223#ref-61); [Walters et al., 2013](https://wellcomeopenresearch.org/articles/5-223#ref-60)) as well as salt water ([Mannas et al., 2014](https://wellcomeopenresearch.org/articles/5-223#ref-41); [Martinez-Urtaza et al., 2004](https://wellcomeopenresearch.org/articles/5-223#ref-42)). Reptiles are often infected by Salmonella ([Corrente et al., 2017](https://wellcomeopenresearch.org/articles/5-223#ref-12); [Kanagarajah et al., 2018](https://wellcomeopenresearch.org/articles/5-223#ref-29); [Mukherjee et al., 2019](https://wellcomeopenresearch.org/articles/5-223#ref-45); [Pulford et al., 2019](https://wellcomeopenresearch.org/articles/5-223#ref-52)), and S. enterica strains can invade plant cells, and survive in soil ([Dyda et al., 2020](https://wellcomeopenresearch.org/articles/5-223#ref-15); [Jechalke et al., 2019](https://wellcomeopenresearch.org/articles/5-223#ref-25); [Schikora et al., 2012](https://wellcomeopenresearch.org/articles/5-223#ref-55)). The degree of overlap between bacterial populations from those sources and those that infect humans and animals has not yet been adequately addressed.” That publication contains 10,000 genomes which were sequenced in part to address this topic, and which were also the source of multiple HC20_373 genomes described here.

4. Both problematical text excerpts have now been changed.
5. Summary. This review exemplifies the disbelief that faced an earlier version of this manuscript which was rejected. We believe that it is worth presenting that information in the summary to acknowledge the controversial aspects of our claims.
6. Phages and plasmids in nature. Blast best hits are now included in Table S4D.
